# Aortic Root Replacement Surgery—A Center Experience with Biological Valve Prostheses

**DOI:** 10.3390/jcdd10030107

**Published:** 2023-03-02

**Authors:** Mohamed Salem, Maximilian Boehme, Christine Friedrich, Markus Ernst, Thomas Puehler, Georg Lutter, Felix Schoeneich, Assad Haneya, Jochen Cremer, Jan Schoettler

**Affiliations:** 1 Department of Cardiovascular Surgery, University Hospital Schleswig-Holstein, Campus Kiel, 24105 Kiel, Germany; 2 Department of Pediatric Cardiac Surgery, Pediatric Cardiac Centre, University Hospital of Gießen and Marburg, Campus Gießen, 35385 Gießen, Germany; 3 DZHK (German Centre for Cardiovascular Research), Partner Site Hamburg/Kiel/Lübeck, Potsdamer Str. 58, 10785 Berlin, Germany

**Keywords:** aortic root surgery, biological aortic prostheses, thoracic aortic diseases

## Abstract

Objective: Outcomes after surgical aortic root replacement using different valved conduits are rarely reported. The present study shows the experience of a single center with the use of the partially biological LABCOR (LC) conduit and the fully biological BioIntegral (BI) conduit. Special attention was paid to preoperative endocarditis. Methods: All 266 patients who underwent aortic root replacement by an LC conduit (*n* = 193) or a BI conduit (*n* = 73) between 01/01/2014 and 31/12/2020 were studied retrospectively. Dependency on an extracorporeal life support system preoperatively and congenital heart disease were exclusion criteria. For patients with (*n* = 67) and without (*n* = 199) preoperative endocarditis subanalyses were made. Results: Patients treated with a BI conduit were more likely to have diabetes mellitus (21.9 vs. 6.7%, *p* < 0.001), previous cardiac surgery (86.3 vs. 16.6%; *p* < 0.001), permanent pacemaker (21.9 vs. 2.1%; *p* < 0.001), and had a higher EuroSCORE II (14.9 vs. 4.1%; *p* < 0.001). The BI conduit was used more frequently for prosthetic endocarditis (75.3 vs. 3.6%; <0.001), and the LC conduit was used predominantly for ascending aortic aneurysms (80.3 vs. 41.1%; <0.001) and Stanford type A aortic dissections (24.9 vs. 9.6%; *p* = 0.006). The LC conduit was used more often for elective (61.7 vs. 47.9%; *p* = 0.043) and emergency (27.5 vs. 15.1%; *p* = 0–035) surgeries, and the BI conduit for urgent surgeries (37.0 vs. 10.9%; *p* < 0.001). Conduit sizes did not differ significantly, with a median of 25 mm in each case. Surgical times were longer in the BI group. In the LC group, coronary artery bypass grafting and proximal or total replacement of the aortic arch were combined more frequently, whereas in the BI group, partial replacement of the aortic arch were combined. In the BI group, ICU length of stay and duration of ventilation were longer, and rates of tracheostomy and atrioventricular block, pacemaker dependence, dialysis, and 30-day mortality were higher. Atrial fibrillation occurred more frequently in the LC group. Follow-up time was longer and rates of stroke and cardiac death were less frequent in the LC group. Postoperative echocardiographic findings at follow-up were not significantly different between conduits. Survival of LC patients was better than that of BI patients. In the subanalysis of patients with preoperative endocarditis, significant differences between the used conduits were found with respect to previous cardiac surgery, EuroSCORE II, aortic valve and prosthesis endocarditis, elective operation, duration of operation, and proximal aortic arch replacement. For patients without preoperative endocarditis, significant differences were observed concerning previous cardiac surgery, pacemaker implantation history, duration of procedure, and bypass time. The Kaplan–Meier curves for the subanalyses showed no significant differences between the used conduits. Conclusions: Both biological conduits studied here are equally suitable in principle for complete replacement of the aortic root in all aortic root pathologies. The BI conduit is often used in bail-out situations, especially in severe endocarditis, without being able to show a clinical advantage over the LC conduit in this context.

## 1. Introduction

Diseases of the aortic root are diverse and sometimes, as in Marfan syndrome, genetically predisposed [1]. Typical pathologies include poststenotic aortic dilatation in the presence of aortic valve stenosis, basal aneurysm, often with concomitant aortic valve insufficiency, and aortic dissection extending to the aortic base, possibly with resulting aortic valve insufficiency [2]. In addition, complete aortic root calcification with aortic valve vitium, e.g., after thoracic radiation therapy, and extensive endocarditis of the aortic valve with involvement of the proximal ascending aorta are included. The indication for total aortic root replacement arises when both the aortic valve and the proximal ascending aorta need replacing for the reasons listed above. Total aortic root replacement was first described by Bentall in 1968 [3]. Reconstruction of the aortic root must be distinguished from aortic root replacement. The techniques according to David or Yacoub are available for this purpose [4,5].

Various industrially manufactured conduits are available for complete replacement of the aortic root. A conduit consists of a tubular prosthesis with an integrated aortic valve prosthesis. There are mechanical conduits that include a mechanical prosthetic heart valve and biological conduits with a biological valve. Increasingly, affected patients prefer the implantation of a biological conduit because they wish to avoid anticoagulant therapy [6].

In addition, from the point of view of cardiac surgery, various aspects favor the implantation of a biological rather than a mechanical conduit. Thus, in the context of endocarditis, in emergency situations, and in complex redo surgery procedures, biological conduits are often favored by surgeons [7]. Therefore, biological conduits are now predominantly used in many cardiac surgery centers. This trend does not seem to have a negative effect on early or late survival [2,8,9].

So far, it has not been sufficiently proven which biological conduit is advantageous with regard to the outcome. The aim of the present study is to report our experience with morbidity and mortality after biological aortic root replacement with a LABCOR conduit, which carries a biological heart valve but the remaining components are synthetic, and a BioIntegral conduit, which is completely made of biological tissue. Special attention was paid to patients with preoperative endocarditis.

## 2. Methods

### 2.1. Patients

All patients who underwent aortic root replacement with a LABCOR or a BioIntegral conduit between 01/01/2014 and 31/12/2020 at the Department of Cardiovascular Surgery, University Hospital Schleswig-Holstein, Campus Kiel, Germany, were studied retrospectively. Patients with congenital heart disease and preoperative insertion of extra corporeal life support were excluded. All included patients had given written informed consent for research with patient data. The study was approved by the local ethics committee of Christian-Albrechts-Universität zu Kiel (D463/21).

### 2.2. Imaging Preoperative Diagnostics

Almost all patients included in the study had received transthoracic echocardiographic assessment preoperatively in the case of an elective procedure. The Patients were examined by transesophageally echocardiography in cases of inadequate transthoracic echocardiography.. In the context of emergency surgery, patients received transesophageal echocardiography immediately after induction of anesthesia. The majority of patients received preoperative thoracic computed tomography, in most cases, with contrast agent. To diagnose or exclude coronary artery disease requiring treatment before surgery, every electively operated patient underwent left heart catheterization as standard. In individual cases, the need for aortic root replacement was determined intraoperatively in the course of other cardiac surgical procedures without specific preoperative diagnostics.

### 2.3. Surgical Technique

After general anesthesia and endo-tracheal intubation, the chest was entered through a median sternotomy. Full heparin administration was followed by institution of cardiopulmonary bypass either through right atrial or through bicaval cannulation to the distal ascending aorta return. An arterial cannula would be advanced into the left ventricle through the right superior pulmonary vein in the presence of type A aortic dissection [10]. In cases of proximal, partial, or complete arch replacement, deep hypothermia was first induced, and the appropriate section of the aorta was then replaced in transient hypothermic circulatory arrest, with selective cerebral perfusion if necessary. In cases without aortic arch surgery, moderate hypothermia was applied after induction of heart fibrillation, followed by cross-clamping of the ascending aorta, and administration of ante- and usually retrograde cardioplegia infusion. In operations with simultaneous coronary surgery, the bypasses were first created in a typical manner. If the mitral valve required attention, we would consider starting with mitral valve surgery. Then, the aortic valve was visualized via a supracoronary approach. The valve leaflets were removed, and the aortic valve annulus was carefully decalcified if necessary. If endocarditis affected the aortic annulus, it would be reconstructed with bovine pericardium. The coronary artery ostia were excised in a circular manner and the non-coronary sinus was resected. After appropriate measurement, the selected conduit would be implanted in the usual manner with single sutures. This was followed by reanastomosis of the coronary vessels with the conduit. Finally, the conduit was anastomosed distally to the ascending aorta or, in case of previous arch replacement, to the tubular prosthesis placed distally through end-to-end. After echocardiographic exclusion of air bubbles in the left ventricle, the cross-clamp was removed and thus reperfusion was started. After rewarming of the patient and echocardiographic assessment of the surgical result, extracorporeal circulation was discontinued. With occasional exceptions for dissections, a graft inclusion was made using the native ascending aorta. This was followed by careful hemostasis, placement of chest drains and pacing wires, and finally wound closure in layers.

### 2.4. Data Collection

Data were collected retrospectively. Pre-, intra- and postoperative variables were taken from the Hospital medical records. Follow-up was performed by mail. All data collected were documented in an anonymized form in an Excel spreadsheet.

### 2.5. Statistical Analyses

First, the overall population was compared with respect to the two conduits. Subanalyses were then performed for the patients with and those without preoperative endocarditis. Characteristics of the patient groups were presented as the median with 25th and 75th percentiles. Non-normally distributed continuous data as well as ordinal data were compared using the Mann-Whitney U test. Normal distribution was assessed using the Kolmogorov–Smirnov test. Categorical data were summarized as absolute (*n*) and relative (%) frequencies and compared by Chi^2^-test or Fisher’s exact test as appropriate. Survival was calculated on right-censored data using Kaplan–Meier analyses and compared for differences between the groups using the log rank test. Pre- and intraoperative variables were assessed for their adjusted impact on early mortality by multivariable logistic regression separately (model 1 and 2). Subsequently, significant pre- and intraoperative risk factors were included in model 3, with a goodness of fit, described by Cox and Snell R-Squared, of 0.204 (Model 1), 0.154 (model 2), and 0.209 (model 3). Variables with less than eight events and EuroSCORE II (The European System for Cardiac Operative Risk Evaluation) were excluded from the multivariable analyses; the latter was excluded since it complicated the detection of single risk factors due to multicollinearity. All tests were performed as two-sided tests and a *p*-value of ≤0.05 was regarded as statistically significant. Data analysis was performed using IBM SPSS Statistics for Windows (Version 28.0).

## 3. Results

Between 01/01/2014 and 12/31/2020, a total of 266 patients underwent full aortic root replacement at the Department of Cardiovascular Surgery at the University Hospital Schleswig-Holstein, Campus Kiel. A total of 73 patients received a BioIntegral conduit and 193 patients received a LABCOR conduit. A total of 67 patients were admitted with preexisting endocarditis and 199 patients without endocarditis.

### 3.1. Total Group

**Figure 1:**
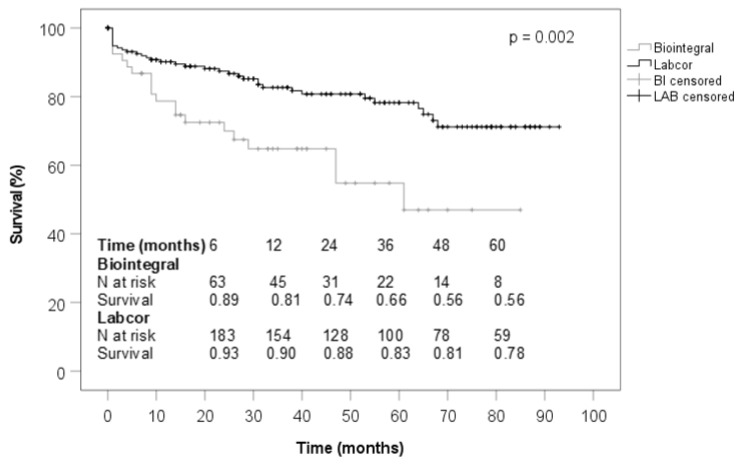
Survival (total).

Table 1 shows the baseline characteristics. Diabetes mellitus, renal insufficiency, prior cardiac surgery with median sternotomy, and pacemaker implantation were more frequently found in the BioIntegral group than in the LABCOR one. The median logistic EuroSCORE II was significantly higher in the BioIntegral collective than in the LABCOR collective (15% vs. 4%). Preoperative aortic root disease, aortic valve stenosis, and prosthetic valve endocarditis were significantly more common in the BioIntegral conduit group, whereas aortic valve regurgitation, Stanford type A aortic dissection, and aneurysm of the ascending aorta were significantly less (Table 2). The intraoperative variables showed that the BioIntegral conduit was implanted less frequently in elective aortic root replacement surgery but more frequently in urgent and emergency surgery. In the BioIntegral collective, operation times were significantly longer, the proportion of combined cardiac surgery was significantly lower, and simultaneous total aortic arch replacement, frozen elephant trunk implantation, or coronary artery bypass grafting were performed less frequently in addition to aortic root replacement. Partial aortic arch replacement occurred more frequently in the BioIntegral group. The conduit sizes used were nearly identical with a median of 25 mm in both groups (Table 3). During postoperative treatment, it was found that patients treated with a BioIntegral conduit required significantly longer treatment in the intensive care unit, with longer mechanically ventilation, as well as they were tracheotomized more frequently. In the BioIntegral group, atrioventricular block and the need for pacemaker implantation was more frequent postoperatively; however, postoperative atrial fibrillation was observed significantly less. The BioIntegral patients suffered from renal insufficiency requiring hemodialysis more often during the postoperative course. The 30-day mortality was significantly higher in the BioIntegral group (26.0% vs. 7.8%) (Table 4). Follow-up time was significantly lower in the BioIntegral group. Mediastinitis and cardiac causes of death were significantly more frequent in the BioIntegral patients. Mortality at the time of follow-up was not significantly different between the two groups, being 28.8% in the BioIntegral group and 18.1% in the LABCOR group (Table 5). Transvalvular pressure gradient, effective valve orifice area, global left ventricular function and rate of conduit valve insufficiency did not diverge significantly (Table 6). Figure 1 shows that the patients treated with a BioIntegral conduit had a significantly worse survival rate than those implanted with a LABCOR conduit.

### 3.2. Endocarditis Group

**Figure 2:**
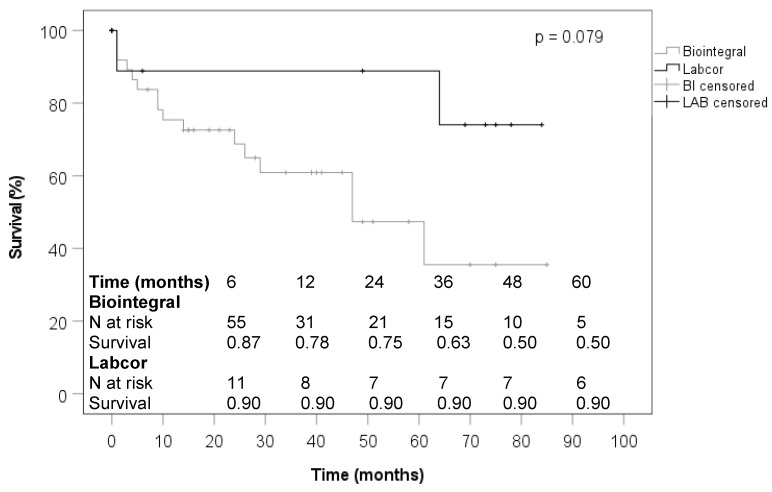
Survival (endocarditis).

In the subanalysis of patients with endocarditis, those who received a BioIntegral conduit were significantly more likely to have the condition after prior cardiac surgery with median sternotomy and their logistic EuroSCORE II was significantly higher at 16% versus 9% median (Table 1). Regarding aortic root pathologies, prosthetic endocarditis was significantly more frequent in the BioIntegral group at 100% versus 58.3%, and endocarditis of the native aortic valve was significantly less frequent at 0% versus 41.7% (Table 2). BioIntegral patients in the endocarditis group underwent elective cardiac surgery significantly more often and the median duration of the operations was significantly longer. In the BioIntegral group, proximal or partial aortic arch replacement was performed significantly more often (Table 3). Regarding postoperative variables, including 30-day mortality, no significant differences were observed between the two conduits in endocarditis patients (Table 4). During the follow-up of endocarditis patients, subsequent prosthetic endocarditis occurred in 7.7% of BioIntegral patients and in none of the LABCOR group, with no statistical difference (Table 5). None of the echocardiographic parameters measured during the follow-up of the endocarditis patients showed a significant difference depending on the implanted conduit (Table 6). As shown in Figure 2, patients receiving a BioIntegral conduit did not experience worse survival compared to those who received a LABCOR conduit.

### 3.3. Non-Endocarditis Group

**Figure 3:**
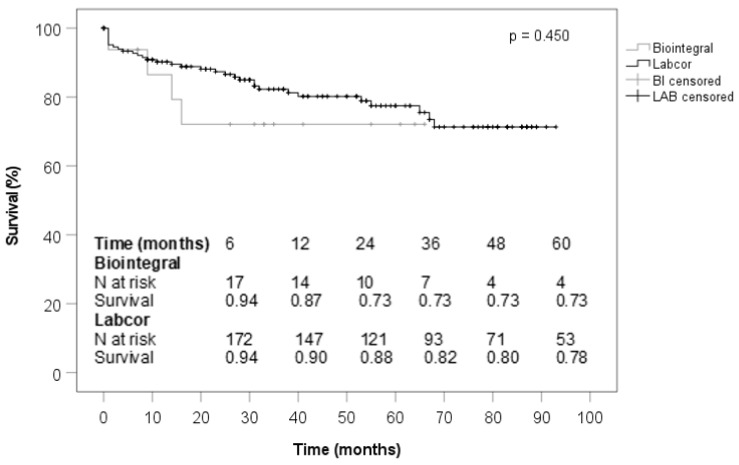
Survival (no endocarditis).

In the Non-Endocarditis group, patients who received a BioIntegral conduit were significantly more likely to have had prior cardiac surgery and they were significantly more likely to have a permanent pacemaker (Table 1). The pathologies of the aortic root were similar (Table 2). Median procedure duration and bypass time were significantly longer in the BioIntegral group (Table 3). Postoperative variables did not differ in any respect in this subanalysis (Table 4). No significant differences were seen with regard to the findings and echocardiographic parameters obtained at follow-up (Table 5 and Table 6). The survival curves of both prosthetic groups were comparable in the subanalysis of patients without preoperative endocarditis (Figure 3).

### 3.4. Adjusted Risk Factors

The multivariable analysis revealed age, female gender, diabetes mellitus, non-paroxysmal atrial fibrillation, renal insufficiency, and emergency admission as preoperative risk factors, while intraoperatively age and female gender were also risk factors as well as endocarditis and bypass time. Combined pre- and intraoperative risk factors were age, female gender, endocarditis, diabetes mellitus, renal insufficiency, and emergency admission. The BioIntegral Conduit was not shown to be a risk factor either preoperatively or intraoperatively (Table 7).

## 4. Discussion

At present, there are no literature data comparing the performance of the LABCOR and BioIntegral conduits in terms of perioperative variables and outcome in patients requiring full aortic root replacement. Therefore, our experience in 266 patients over a 5-year period may contribute to shed some lights on the subject since our initial report in 2018 of the first 33 patients treated with a BioIntegral conduit in our hospital [11]. The LABCOR conduit consists of a stented porcine valve prosthesis and a synthetic tubular graft, whilst the BioIntegral conduit is a bovine pericardial tube including a stentless porcine valve. As stated by Puehler et al., reconstructive surgery of the aortic root seems more popular compared to root replacement due to infection, bleeding, and reoperation rate [11]. Additionally, reconstruction of the aortic root is considered the gold standard in the case of valve anomalies [12,13,14]. The choice between both conduits in cases of aortic root replacement is the responsibility of the respective cardiac surgeon; however, there is an internal consensus to use the fully biological BioIntegral conduit in cases with endocarditis [9]. Homografts or autografts were not used for total aortic root replacement during the study period at our department [15].

The current analysis showed that the LABCOR conduit was used more frequently during the study period and that the patients who were treated with a BioIntegral conduit had a worse preoperative status, and they suffered from more complications during the early postoperative course and had a higher mortality rate. Echocardiographic findings were comparable at the time of follow-up between both conduits used, with no significant differences during the study period. Wendt et al. analyzed the mid-term hemodynamic performance of BioIntegral and BioValsalva conduits after aortic root replacement in a smaller cohort with 55 patients [16]. Like the LABCOR conduit, the BioValsalva conduit is composed of a porcine heart valve and a synthetic tube. The most important difference is that the BioValsalva conduit, in contrast to the LABCOR conduit, carries a stentless valve prosthesis. Wendt and colleagues did not observe significant differences between the BioIntegral and the BioValsalva conduit with regard to the hemodynamic measurements at follow-up. The pressure gradients for all valve sizes in their group treated with a BioIntegral conduit were unremarkable and similar to the transvalvular gradients determined in our BioIntegral group. In comparison to the work of Wendt and coworkers, both of our patient groups appeared significantly sicker and suffered from significantly higher mortality. For example, in our patients who were treated with a BioIntegral conduit, the median EuroSCORE II was 14.9%, the rate of previous cardiac surgery procedures was 86.3%, and 30-day mortality was 26.0%, which is higher than in patients of Wendt and coauthors who also received a BioIntegral conduit. Wendt and coworkers recorded a median EuroSCORE II of only 5.3% and a low rate of previous cardiac surgery of 10.7%in their BioIntegral group. Correlating to the relatively low risk profile of their patients, the 30-day mortality in their BioIntegral group was just 3.6%. It is uncertain whether they provided their sicker patients with another conduit or possibly also with homografts or autografts. However, the discrepancy between the groups in Kiel and Essen can also be explained by the strikingly low rate of patients with existing endocarditis who underwent aortic root replacement during the period studied there. Wendt et al. treated only two patients (3.6%) with preoperative endocarditis and both patients received a BioValsalva conduit.

In our analysis, 25.2% patients suffered from active infective endocarditis prior to total aortic root replacement and underwent subanalysis. Additionally, in this high-risk group, all patients received a BioIntegral or LABCOR conduit without exception. Comparability between the two groups was more favorable in this subanalysis, and preoperative morbidity was high in each case but was more homogeneously distributed than in the overall comparison between both groups. Nevertheless, the patients who received a BioIntegral conduit were still more complex, with 96.4% having previous cardiac surgery via a median sternotomy and 100% having preoperative prosthetic endocarditis after aortic valve or aortic root replacement or after transcatheter aortic valve implantation in individual cases. In the LABCOR group, the proportion of patients with prior surgery and preoperative prosthetic infection was still high, but significantly lower at 58.3% each. Considering only cases with preexisting endocarditis, the BioIntegral conduit was not significantly different from the LABCOR conduit in terms of subsequent prosthetic endocarditis in our study, at 7.7% versus 0%. Furthermore, no significant differences were observed in this subanalysis in either numerical echocardiographic values or dichotomous rates of conduit valve regurgitation during follow-up. In this high-risk population, Kaplan–Meier analysis did not show a significant survival advantage for either conduit. Our subanalysis on patients without preoperative endocarditis reported a better outcome than in the total or endocarditis groups, with no significant differences between the two conduits, although again the BioIntegral group was worse from baseline with significantly more prior cardiac surgeries and pacemaker implantation and longer operative and bypass times.

In this high-risk endocarditis population, the 30-day mortality was 30.9% for BioIntegral versus 16.7% for LABCOR without a significant difference. A study from Heinz et al. on patients with severe destructive aortic root endocarditis showed that the Freestyle root replacement was used successfully with no technical complications in such cases. In view of this complex patient population, short- and long-term results make this conduit a reliable choice for treatment of this condition [17]. Their 30-day mortality was 19.4% (*n* = 6) which is comparable to our LABCOR mortality of 16.7%. The actuarial survival at 5 and 10 years was 61.9% and 54.2%, respectively. Freedom from death, reoperation for prostheses dysfunction, and recurrence of endocarditis as the composite end points at 5 and 10 years was 56.3% and 53.1%, respectively.

In summary, total aortic root replacement, especially as a reoperation, is one of the most complex cardiac surgical procedures. The outcome of operated patients depends mainly on concomitant diseases, but especially on preexisting endocarditis, irrespective of the conduit used. Although our results should be interpreted with caution due to the different risk profiles of both groups, the BioIntegral group was sicker in several aspects, and we conclude that even in bail-out situations, such as extensive aortic valve or prosthesis endocarditis, a purely biological conduit does not necessarily have to be implanted, but that the only partially biological LABCOR conduit can also be used in these situations without tangible disadvantages.

## 5. Limitations

The studies were performed retrospectively. Thus, randomization was excluded and the proportion of patients who received a LABCOR conduit was significantly higher. The patients who were treated with a BioIntegral conduit during the period under study showed a significant morbidity in the overall comparison and the corresponding surgical procedures were significantly more complex due to the difficulty of the tissue dissection due to redo surgery.

## 6. Conclusions

Both conduits with a biological valve prosthesis for complete aortic root replacement compared with each other showed a good overall performance. The fully biological BioIntegral Conduit is more often chosen in situations with pre-existing endocarditis due to concerns that synthetic materials may promote reinfection. However, valid arguments against the use of the semisynthetic LABCOR conduit even in patients with preoperative extensive endocarditis in the region of the aortic root were not found in our studies.

## Figures and Tables

**Table 1:** Baseline characteristics.

Variables	Subgroups	BioIntegral (*n* = 73)	LABCOR (*n* = 193)	*p*
Age (years)	total	69.0 (59.0; 75.0)	66.0 (57.0; 74.5)	0.228
	endocarditis	71.0 (60.0; 76.0)	62.5 (52.8; 71.5)	0.067
	no endocarditis	67.5 (54.5; 73.3)	66.0 (57.0; 75.0)	0.699
Male (%)	total	64.4	68.4	0.534
	endocarditis	63.6	83.3	0.310
	no endocarditis	66.7	67.4	0.949
Body mass index	total	25.9 (23.7; 28.7)	27.2 (24.5; 29.9)	0.119
	endocarditis	25.9 (23.6; 28.7)	28.6 (25.2; 34.0)	0.116
	no endocarditis	26.7 (23.8; 30.2)	27.2 (24.4; 29.7)	0.891
Arterial hypertension (%)	total	76.7	67.9	0.159
	endocarditis	81.8	58.3	0.121
	no endocarditis	61.1	68.5	0.522
Hyperlipidemia (%)	total	34.2	27.5	0.278
	endocarditis	32.7	33.3	1.000
	no endocarditis	38.9	27.1	0.288
Diabetes mellitus (%)	total	21.9	6.7	<0.001
	endocarditis	23.6	0.0	0.104
	no endocarditis	16.7	7.2	0.164
Smoker (%)	total	21.9	19.2	0.617
	endocarditis	20.0	33.3	0.444
	no endocarditis	27.8	18.2	0.347
Chronic obstructive lung disease (%)	total	12.3	11.9	0.927
	endocarditis	12.7	16.7	0.658
	no endocarditis	11.1	11.6	1.000
Pulmonary hypertension (%)	total	9.6	8.8	0.843
	endocarditis	9.1	0.0	0.576
	no endocarditis	11.1	9.4	0.684
Renal insufficiency (%)	total	35.6	22.8	0.034
	endocarditis	43.6	41.7	0.901
	no endocarditis	11.1	21.5	0.376
Dialysis (%)	total	2.7	2.6	1.000
	endocarditis	1.8	8.3	0.328
	no endocarditis	5.6	2.2	0.381
Peripheral artery disease (%)	total	1.4	2.1	1.000
	endocarditis	0.0	16.7	0.030
	no endocarditis	5.6	1.1	0.249
Malignom in history (%)	total	9.6	12.4	0.519
	endocarditis	10.9	8.3	1.000
	no endocarditis	5.6	12.7	0.703
Stroke in history (%)	total	8.2	5.2	0.389
	endocarditis	9.1	0.0	0.576
	no endocarditis	5.6	5.5	1.000
Coronary heart disease (%)	total	46.6	40.4	0.364
	endocarditis	52.7	66.7	0.379
	no endocarditis	27.8	38.7	0.363
Previous myocardial infarction (%)	total	9.6	9.8	0.950
	endocarditis	9.1	8.3	1.000
	no endocarditis	11.1	9.9	0.699
Previous coronary stenting (%)	total	13.7	9.8	0.368
	endocarditis	16.4	16.7	1.000
	no endocarditis	5.6	9.4	1.000
Previous cardiac surgery (%)	total	86.3	16.6	<0.001
	endocarditis	96.4	58.3	0.001
	no endocarditis	55.6	13.8	<0.001
Pacemaker (%)	total	21.9	2.1	<0.001
	endocarditis	23.6	0.0	0.104
	no endocarditis	16.7	2.2	0.017
Paroxysmal atrial fibrillation (%)	total	20.5	16.1	0.388
	endocarditis	21.8	8.3	0.435
	no endocarditis	16.7	16.6	1.000
Non-paroxysmal atrial fibrillation (%)	total	11.0	12.4	0.741
	endocarditis	14.5	16.7	1.000
	no endocarditis	0.0	12.2	0.230
EuroSCORE II (%)	total	14.9 (7.1; 25.0)	4.1 (1.6; 12.0)	<0.001
	endocarditis	16.1 (9.6; 27.5)	9.1 (3.3; 20.8)	0.050
	no endocarditis	5.3 (3.3; 17.1)	3.9 (1.5; 12.0)	0.115

**Table 2:** Pathologies of the aortic root.

Variables	Subgroups	BioIntegral (*n* = 73)	LABCOR (*n* = 193)	*p*
Aortic valve stenosis (%)	total	41.1	25.4	0.012
	endocarditis	40.0	33.3	0.753
	no endocarditis	44.4	24.9	0.093
Aortic valve insufficiency (%)	total	52.1	74.1	<0.001
	endocarditis	49.1	58.3	0.562
	no endocarditis	61.1	75.1	0.258
Aortic valve endocarditis (%)	total	0.0	2.6	0.327
	endocarditis	0.0	41.7	<0.001
	no endocarditis	0.0	0.0	1.000
Prosthesis endocarditis (%)	total	75.3	3.6	<0.001
	endocarditis	100.0	58.3	<0.001
	no endocarditis	0.0	0.0	1.000
Aortic dissection Stanford type A (%)	total	9.6	24.9	0.006
	endocarditis	0.0	0.0	1.000
	no endocarditis	38.9	26.5	0.276
Ascending aorta aneurysm (%)	total	41.1	80.3	<0.001
	endocarditis	30.9	41.7	0.510
	no endocarditis	72.2	82.9	0.331

**Table 3:** Intraoperative Variables.

Variables	Subgroups	BioIntegral (*n* = 73)	LABCOR (*n* = 193)	*p*
Elective operation (%)	total	47.9	61.7	0.043
	endocarditis	47.3	8.3	0.020
	no endocarditis	50.0	65.2	0.201
Urgent operation (%)	total	37.0	10.9	<0.001
	endocarditis	41.8	66.7	0.118
	no endocarditis	22.2	7.2	0.053
Emergency operation (%)	total	15.1	27.5	0.035
	endocarditis	10.9	25.0	0.345
	no endocarditis	27.8	27.6	1.000
Conduit size (mm)	total	25.0 (23.0; 27.0)	25.0 (23.0; 27.0)	0.530
	endocarditis	25.0 (23.0; 27.0)	23.0 (23.0; 25.0)	0.383
	no endocarditis	25.0 (23.0; 27.5)	25.0 (23.0; 27.0)	0.935
Duration of procedure (minutes)	total	415.0 (364.5; 486.0)	312.0 (260.5; 362.5)	<0.001
	endocarditis	427.0 (372.0; 494.0)	366.5 (302.5; 449.5)	0.039
	no endocarditis	390.0 (301.0; 472.5)	307.0 (258.0; 359.5)	0.001
Bypass time (minutes)	total	249.0 (203.5; 277.0)	195.0 (162.0; 232.0)	<0.001
	endocarditis	250.0 (213.0; 280.0)	220.0 (188.0; 245.0)	0.109
	no endocarditis	215.5 (184.0; 277.0)	195.0 (160,0; 232.0)	0.028
Cross-clamp time (minutes)	total	170.0 (145.5; 199.0)	139.0 (114.0; 169.5)	<0.001
	endocarditis	177.0 (146.0; 202.0)	166.5 (146.3; 193.0)	0.862
	no endocarditis	152.5 (139.5; 172.3)	137.0 (113.0; 168.5)	0.135
Combined cardiac surgery (%)	total	54.8	68.4	0.038
	endocarditis	52.7	50.0	0.864
	no endocarditis	61.1	69.6	0.458
Proximal aortic arch replacement (%)	total	67.1	72.5	0.385
	endocarditis	65.5	100.0	0.014
	no endocarditis	72.2	70.7	0.893
Partial aortic arch replacement (%)	total	32.9	19.7	0.023
	endocarditis	34.5	0.0	0.014
	no endocarditis	27.8	21.0	0.549
Complete aortic arch replacement (%)	total	0.0	7.8	0.014
	endocarditis	0.0	0.0	1.000
	no endocarditis	0.0	8.3	0.370
Frozen elephant trunk (%)	total	0.0	2.1	0.578
	endocarditis	0.0	0.0	1.000
	no endocarditis	0.0	2.2	1.000
Mitral valve replacement (%)	total	2.7	2.6	1.000
	endocarditis	3.6	8.3	0.452
	no endocarditis	0.0	2.2	1.000
Mitral valve reconstruction (%)	total	6.8	5.2	0.564
	endocarditis	9.1	8.3	1.000
	no endocarditis	0.0	5.0	1.000
Tricuspid valve reconstruction (%)	total	1.4	0.0	0.274
	endocarditis	1.8	0.0	1.000
	no endocarditis	0.0	0.0	1.000
Coronary bypass surgery (%)	total	6.8	21.8	0.004
	endocarditis	5.5	25.0	0.066
	no endocarditis	11.1	21.5	0.376
Closure of persistent foramen ovale (%)	total	5.5	5.7	1.000
	endocarditis	7.3	8.3	1.000
	no endocarditis	0.0	5.5	0.604
Closure of atrial septum defect (%)	total	0.0	1.0	1.000
	endocarditis	0.0	0.0	1.000
	no endocarditis	0.0	1.1	1.000
Closure of left atrial appendix (%)	total	1.4	4.1	0.452
	endocarditis	1.8	0.0	1.000
	no endocarditis	0.0	4.4	1.000
Left atrial ablation (%)	total	0.0	2.6	0.327
	endocarditis	0.0	0.0	1.000
	no endocarditis	0.0	2.8	1.000
Simultaneous thrombectomy of carotid artery (%)	total	2.7	0.5	0.184
	endocarditis	3.6	0.0	1.000
	no endocarditis	0.0	0.6	1.000

**Table 4:** Postoperative Variables.

Variables	Subgroups	BioIntegral (*n* = 73)	LABCOR (*n* = 193)	*p*
Stay on intensive care unit (days)	total	6.0 (3.0; 9.5)	3.0 (2.0; 8.0)	0.003
	endocarditis	6.0 (3.0; 10.0)	4.5 (2.3; 9.3)	0.367
	no endocarditis	6.0 (2.0; 9.5)	3.0 (2.0; 8.0)	0.165
Duration of ventilation (hours)	total	31.0 (20.0; 162.0)	18.0 (12.0; 64.0)	<0.001
	endocarditis	46.0 (22.0; 174.0)	30.5 (12.8; 116.8)	0.386
	no endocarditis	21.5 (13.0; 72.5)	18.0 (12.0; 63.0)	0.517
Tracheotomy (%)	total	17.8	8.8	0.038
	endocarditis	20.0	8.3	0.678
	no endocarditis	11.1	8.8	0.670
Stroke (%)	total	4.1	8.3	0.237
	endocarditis	3.6	0.0	1.000
	no endocarditis	5.6	8.8	1.000
Delir (%)	total	20.5	22.3	0.760
	endocarditis	18.2	16.7	1.000
	no endocarditis	27.8	22.7	0.571
Atrial fibrillation (%)	total	26.0	44.0	0.007
	endocarditis	21.8	33.3	0.460
	no endocarditis	38.9	44.8	0.633
Atrioventricular block (%)	total	23.3	10.4	0.007
	endocarditis	27.3	50.0	0.171
	no endocarditis	11.1	7.7	0.643
Pacemaker dependency (%)	total	19.2	7.8	0.008
	endocarditis	23.6	33.3	0.483
	no endocarditis	5.6	6.1	1.000
Dialysis (%)	total	27.4	12.4	0.003
	endocarditis	30.9	25.0	1.000
	no endocarditis	16.7	11.6	0.461
Redo surgery due to bleeding (%)	total	26.0	17.1	0.101
	endocarditis	27.3	16.7	0.716
	no endocarditis	22.2	17.1	0.529
Impaired wound healing (%)	total	19.2	15.5	0.477
	endocarditis	23.6	33.3	0.483
	no endocarditis	5.6	14.4	0.477
Hospital stay (days)	total	13.0 (8.5; 21.0)	11.0 (8.0; 17.5)	0.363
	endocarditis	13.0 (8.0; 22.0)	11.5 (5.3; 18.3)	0.534
	no endocarditis	12.5 (9.0; 17.3)	11.0 (8.0; 17.5)	0.405
30-day mortality (%)	total	26.0	7.8	<0.001
	endocarditis	30.9	16.7	0.485
	no endocarditis	11.1	7.2	0.631

**Table 5:** Follow-up.

Variables	Subgroups	BioIntegral (*n* = 73)	LABCOR (*n* = 193)	*p*
Follow-up time (months)	total	14.5 (0.0; 39.0)	31.0 (11.0; 62.5)	<0.001
	endocarditis	12.0 (0.0; 39.0)	56.5 (0.3; 74.5)	0.082
	no endocarditis	28.5 (7.0; 44.5)	31.0 (11.5; 60.0)	0.207
New onset of atrial fibrillation (%)	total	20.8	11.7	0.115
	endocarditis	20.5	11.1	1.000
	no endocarditis	21.4	11.8	0.389
Myocardial infarction (%)	total	3.8	4.7	1.000
	endocarditis	2.6	0.0	1.000
	no endocarditis	7.1	5.0	0.600
Mediastinitis (%)	total	22.6	6.3	0.001
	endocarditis	30.8	0.0	0.088
	no endocarditis	0.0	6.7	1.000
Prosthetic endocarditis (%)	total	7.5	11.6	0.413
	endocarditis	7.7	0.0	1.000
	no endocarditis	7.1	12.5	1.000
Redo cardiac surgery procedure (%)	total	11.3	17.8	0.276
	endocarditis	10.3	11.1	1.000
	no endocarditis	14.3	18.3	1.000
Re-conduit replacement (%)	total	5.7	14.7	0.088
	endocarditis	5.1	11.1	0.472
	no endocarditis	7.1	15.0	0.691
Transcatheter aortic valve implantation (%)	total	1.9	0.0	0.295
	endocarditis	2.5	0.0	1.000
	no endocarditis	0.0	0.0	1.000
Stroke (%)	total	13.2	3.9	0.043
	endocarditis	15.4	0.0	0.571
	no endocarditis	7.1	4.2	0.494
Mortality during follow-up (%)	total	28.8	18.1	0.058
	endocarditis	30.9	16.7	0.485
	no endocarditis	22.2	18.2	0.750
Cardiac cause of death (%)	total	37.7	15.6	0.001
	endocarditis	43.6	11.1	0.125
	no endocarditis	21.4	16.0	0.702

**Table 6:** Echocardiographic findings.

Variables	Subgroups	BioIntegral (*n* = 73)	LABCOR (*n* = 193)	*p*
Left ventricular ejection fraction (%)	total	55.5 (47.0; 63.1)	58.8 (50.0; 64.8)	0.466
	endocarditis	50.0 (37.0; 62.5)	60.0 (60.0; 70.0)	0.141
	no endocarditis	60.0 (55.0; 65.0)	57.5 (50.0; 64.5)	0.498
Conduit valve Pmax (mmHg)	total	17.0 (13.0; 18.8)	19.0 (13.5; 26.3)	0.389
	endocarditis	16.0 (12.5; 17.0)	18.5 (9.0; 32.0)	0.204
	no endocarditis	18.4 (16.0; 39.3)	19.0 (13.0; 24.0)	0.733
Conduit valve Pmean (mmHg)	total	9.0 (8.0; 19.0)	11.0 (9.0; 15.0)	0.345
	endocarditis	8.0 (7.3; 13.0)	9.5 (9.0; 13.8)	0.244
	no endocarditis	10.0 (8.0; 20.0)	11.0 (9.0; 15.5)	0.768
Conduit valve orifice area (cm^2^)	total	1.7 ± 0.64	1.6 ± 0.38	0.697
	endocarditis	1.7 ± 0.56	1.7 ± 0.60	0.968
	no endocarditis	1.9 ± 0.91	1.6 ± 0.26	0.653
Conduit valve insufficiency trace (%)	total	7.4	7.2	1.000
	endocarditis	5.9	0.0	1.000
	no endocarditis	10.0	7.7	0.584
Conduit valve insufficiency severe (%)	total	0.0	1.2	1.000
	endocarditis	0.0	0.0	1.000
	no endocarditis	0.0	1.3	1.000

**Table 7:** Logistic regression analysis on pre- and intraoperative risk factors for 30-day mortality.

Variables	Odds Ratio	95% CI	*p*
**Model 1: Preoperative factors**			
BioIntegral	1.635	0.375–7.125	0.513
Endocarditis	3.503	0.807–15.201	0.094
Age (years)	1.056	1.002–1.112	0.041
Female gender	2.543	1.069–6.051	0.035
Diabetes mellitus	3.253	1.128–9.381	0.029
Non-paroxysmal AF	3.026	1.002–9.137	0.050
Renal insufficiency	2.655	1.076–6.555	0.034
Emergency	3.335	1.230–9.040	0.018
**Model 2: Intraoperative factors**			
BioIntegral	1.245	0.355–4.368	0.732
Endocarditis	3.607	1.021–12.738	0.046
Age (years)	1.091	1.037–1.148	<0.001
Female gender	3.017	1.331–6.838	0.008
Bypass time (min)	1.006	1.001–1.011	0.029
**Model 3: Pre- and intraoperative factors (Model 1 + 2) combined**			
BioIntegral	1.534	0.355–6.627	0.566
Endocarditis	3.231	0.757–13.796	0.113
Age (years)	1.059	1.004–1.117	0.035
Female gender	2.642	1.103–6.327	0.029
Diabetes mellitus	3.187	1.099–9.241	0.033
Non-paroxysmal atrial fibrillation	2.770	0.904–8.491	0.075
Renal insufficiency (creatinine > 120 µmol/L)	2.549	1.027–6.327	0.044
Emergency	3.102	1.133–8.495	0.028
Bypass time (min)	1.004	0.998–1.006	0.176

AF, Atrial fibrillation, Renal insufficiency (creatinine > 120 µmol/L).

## Data Availability

All included patients had given written informed consent for research with patient data.
